# Immune Checkpoints, a Novel Class of Therapeutic Targets for Autoimmune Diseases

**DOI:** 10.3389/fimmu.2021.645699

**Published:** 2021-04-21

**Authors:** Yujia Zhai, Reza Moosavi, Mingnan Chen

**Affiliations:** Department of Pharmaceutics and Pharmaceutical Chemistry, University of Utah, Salt Lake City, UT, United States

**Keywords:** immune checkpoints, autoimmune diseases, therapeutics, fusion protein, viral protein, nucleic acid, cell

## Abstract

Autoimmune diseases, such as multiple sclerosis and type-1 diabetes, are the outcomes of a failure of immune tolerance. Immune tolerance is sustained through interplays between two inter-dependent clusters of immune activities: immune stimulation and immune regulation. The mechanisms of immune regulation are exploited as therapeutic targets for the treatment of autoimmune diseases. One of these mechanisms is immune checkpoints (ICPs). The roles of ICPs in maintaining immune tolerance and hence suppressing autoimmunity were revealed in animal models and validated by the clinical successes of ICP-targeted therapeutics for autoimmune diseases. Recently, these roles were highlighted by the clinical discovery that the blockade of ICPs causes autoimmune disorders. Given the crucial roles of ICPs in immune tolerance, it is plausible to leverage ICPs as a group of therapeutic targets to restore immune tolerance and treat autoimmune diseases. In this review, we first summarize working mechanisms of ICPs, particularly those that have been utilized for therapeutic development. Then, we recount the agents and approaches that were developed to target ICPs and treat autoimmune disorders. These agents take forms of fusion proteins, antibodies, nucleic acids, and cells. We also review and discuss safety information for these therapeutics. We wrap up this review by providing prospects for the development of ICP-targeting therapeutics. In summary, the ever-increasing studies and results of ICP-targeting of therapeutics underscore their tremendous potential to become a powerful class of medicine for autoimmune diseases.

## Introduction

The immune system is powerful and versatile in protecting the body: it neutralizes invading pathogens and toxins, detects and destroys infected and malignant cells, and orchestrates the recovery of compromised tissues. As famously said in the movie Spider-Man, “With great power comes great responsibility.” The immune system has the “responsibility” to refrain from excessively attacking and destroying cells and tissues, particularly self and benign cells and tissues. Immunostasis is the immune system’s adaptation to provide restraint. Immunostasis is achieved and maintained through the competition and interplays between two clusters of activities, immune stimulation and immune regulation. The stimulation wing of immunity ensures the immune system is powerful and specific when immunity is needed to decisively eliminate invading pathogens and malignant cells. Stimulation also cultivates immune memory so that the immune system is able to stem the outgrowth of pathogens or malignance faster and more effectively when it encounters pathogens and malignancy again. The regulation wing of immunity, on the other hand, keeps all the traits or strengths of stimulation in check. As put by a Chinese idiom, 过犹不及(Guò-yóu-bù-jí) or in English, “Going beyond the limit is as bad as falling short,” it is extremely important for the immune system and immune stimulation to stay in check because excessive attack, whether in the sense of target ranges, intensity, or duration, will do more harm than benefit to the body. In addition, excessive attack wastes the energy of the immune system, a loss which could prevent the immune system to use its full strength to tackle real threats. Immune regulatory activities may lead to an outcome termed immune tolerance. Indeed, the immune system has diversified and sophisticated mechanisms to achieve and maintain immune tolerance, ranging from central tolerance, peripheral tolerance, to clonal tolerance (1). The failure of immune tolerance, either due to inherited genetic deficiencies or exogeneous stimulants, is the fundamental reason for autoimmune disorders including type 1 diabetes (T1D), multiple sclerosis (MS), rheumatoid arthritis (RA), and so on. Collectively, these diseases affect approximately 12.5% of world population (2).

**Figure 1:**
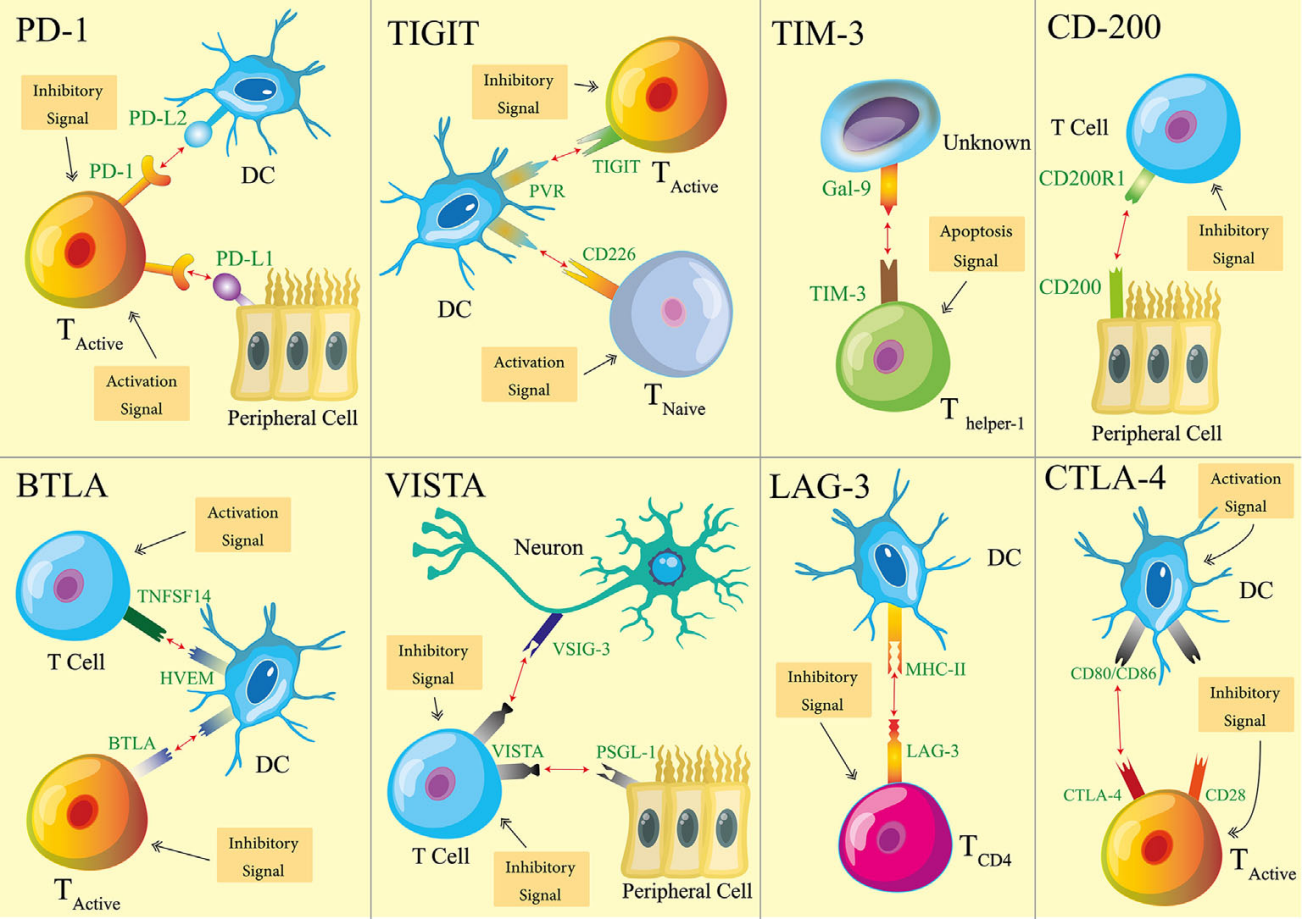
Schematics of eight immune checkpoints (ICPs). For each ICP, one type of cells is used as the representatives that host ICP receptors or ligands. The main immune activation and inhibition implications of ICPs are illustrated with the representative cell types. It is noteworthy that the receptors and ligands may be expressed by additional cell types. The functional implications of ICPs are not limited to what are illustrated here.

One relatively newly discovered mechanism to help to maintain immune tolerance is immune checkpoints (ICPs) (3–6). The first ICP, cytotoxic T lymphocyte antigen-4 (CTLA-4), was reported in the early 1990s (7). In the last 20 plus years, ICPs have proven their importance to immune tolerance thanks to extensive research efforts and consequent discoveries around it. ICPs usually required two biomolecular components, a receptor and a ligand. Receptors are mostly expressed on the surface of immune cells (8–13). Ligands, on the other hand, are expressed sometimes by immune cells, and sometimes by non-hemopoietic cells (14–21). When a receptor and a ligand of an ICP engage with each other, that ICP is flipped on and transmits inhibitory signals. Meanwhile, the cells possessing ICP receptors transform into their immune regulative (suppressive) mode. To date, several ICPs have been revealed. Among them, eight ICPs (**Figure 1**) have been recognized with sufficient knowledge so that experimental therapeutics are developed on the basis of these ICPs. Thus, our review focuses on these eight ICPs: programmed death-1 (PD-1) (22), CTLA-4 (7), B and T cell lymphocyte attenuator (BTLA) (23), T-cell immunoglobulin domain and mucin domain-containing molecule-3 (TIM-3) (24), T cell immunoglobulin and ITIM domain (TIGIT) (25), V-domain Ig suppressor of T cell activation (VISTA) (26), lymphocyte activation gene 3 (LAG-3) (27), and CD200 (28).

The necessity of ICPs in maintaining immune tolerance and preventing autoimmune disorders are underscored by data from animal models. As shown in **Table 1**, when receptors of ICPs are knocked out in mice, autoimmune disorders emerge and manifest in varied severity and organ-specificity, depending the type of ICPs and the genetic background of the mice. The PD-1 receptor knockout increases the prevalence of T1D among nonobese diabetic (NOD) mice (29), results in the lupus-like phenotype in the C57BL/6 background (30), and causes lethal myocarditis among Murphy Roths Large mice (31). The knockout of the BTLA receptor triggers experimental autoimmune encephalitis (EAE), a murine version of MS (23). The knockout of the TIGIT receptor increase susceptibility to EAE (32). The knockout of the LAG-3 receptor exacerbates T1D among NOD mice (33), and renders B6.SJL mice more susceptible to the hyper production of autoantibodies (34). The most striking and pervasive impact of the ICP knockout comes from the knockout of CTLA-4 ICP. Mice carrying such a knockout experience massive lymphoproliferation and die of multiorgan autoimmune destruction within 4 weeks after their birth (35). Recently, we examined the role of the PD-1 ICP and more specifically, the role of PD-1 positive cells in driving autoimmune disorders including EAE and T1D. The depletion PD-1 positive cells drastically delayed the onset of T1D and promoted the recovery from clinical presentations of EAE (36). These results unambiguously demonstrate the central role of PD-1 positive cells in these autoimmune diseases.

**Table 1:** Autoimmune disorders caused by the knockout of ICP molecules.

Modulation of gene	Preclinical model	Autoimmune disorders	Reference
PD-1 knockout	NOD mice	T1D	(29)
	C57BL/6 mice	Lupus-like Phenotype	(30)
	Murphy Roths Large mice	Lethal Myocarditis	(31)
BTLA knockout	BALB/c mice	EAE	(23)
TIGIT knockout	C57BL/6 mice	EAE	(32)
LAG-3 knockout	NOD mice	T1D	(33)
	B6.SJL mice	Hyper Production of Autoantibodies	(34)
CTLA-4 knockout	BALB/c and C57BL/6 mice	Massive Lymphoproliferation and Lethal Multiorgan Destruction	(35)

The necessity of ICPs for immunostasis and immune tolerance is also supported by human data. First and foremost, a therapeutic that reinforces the CTLA-4 ICP, Abatacept (Orencia®), has been approved to treat adult RA. From another perspectives, when the CTLA-4 and PD-1 ICPs are blocked in cancer patients, some patients experienced adverse autoimmune syndromes such as arthralgia, arthritis, Sjögren’s syndrome, and encephalitis (37, 38). The blockade inhibits the function of ICPs, which unleashes the attacking power of immune cells that express the corresponding ICP receptors. Thus, the fact that the ICP blockade induces autoimmune disorders in human proves the suppressive function of ICPs in autoimmunity. Interestingly, the blockade of the CTLA-4 ICP causes significantly more prevalent toxicity than the blockade of PD-1 ICP (39), which echoes the observations of the ICP knockout data from animal models.

With the recognition of the critical role of ICPs in immune tolerance and autoimmune diseases, autoimmune disease therapeutics that target ICPs have already been widely explored. These therapeutics can be generally classified into two categories in terms of strategies: one, enhancement of ICPs; and two, depletion of immune cells that express ICP receptors. We will summarize the therapeutics in these two categories, with an emphasis on their design, mode of action, and efficacy. Before we dive into these therapeutics, we will first provide mechanistic information for eight ICPs that have been exploited as therapeutic targets. **Table 2** summarizes the receptors and ligands of these ICPs.

**Table 2:** Receptors and ligands of immune checkpoints (ICPs).

ICP receptor	Expression pattern of receptors	ICP ligand	Expression pattern of ligands
PD-1	Activated T cell B cell DC Macrophage	PD-L1	Antigen presenting cell Non-hematopoietic cell
		PD-L2	
CTLA-4	Activated T cell DC	CD80	Antigen presenting cell
		CD86	
BTLA	Activated T cell Resting B cell Mature DC NK cells	HVEM	T cell Macrophage DC
TIGIT	Activated T cell NK cell	PVR	DC
		PVRL2	
		PVRL3	
VISTA / PD-1H	T cell NK cell DC Macrophage	VSIG-3	Neuron Glial cell
		PSGL-1	T cell B cell Macrophage DC
TIM-3	T cell DC NK cell Macrophage	Gal-9	
		Phosphatidyl serine	
		High mobility group protein B1	
		Ceacam-1	
LAG-3	Activated T cell B cell DC NK cell	MHC class II	Antigen presentation cell
CD200R	T cell B cell Resting mast cell DC	CD200	Epithelial cell Endothelial cell Lymphoid cell Myeloid cell Neuron

## Mechanisms of Common Immune Checkpoints

For the PD-1 ICP, its receptor, PD-1 is primarily expressed on T cells, B cells, dendritic cells (DCs), and macrophages after their activation. PD-1 has two ligands, PD-L1 and PD-L2. PD-L1 is expressed on antigen presenting cells (APCs) including DCs, macrophages, and B cells (14). PD-L1 is also expressed on non-hematopoietic cells, such as vascular endothelial cells, epithelial cells, fibroblastic reticular cells, and pancreatic islet cells (15). PD-L2, similar to PD-L1, is expressed on both APCs and nonhematopoietic cells such as respiratory tract epithelial cells (16). Regarding PD-1 on T cells, the engagement of PD-1 with its ligands confers inhibitory signals to the T cells that express PD-1. The activation of the PD-1 ICP dampens the proliferation, cytokine secretion, as well as the cytotoxicity of T cells. Regarding PD-1 on human macrophages, an elevated expression of PD-1 by these cells leads to an increase of the IL-10 level and decreases the IL-12 level in blood, a sign of immune self-regulation on macrophages (40, 41). Lastly, PD-1 positive APCs show a weaker ability than PD-1 negative APCs in terms of promoting the differentiation of CD4 T cells into T regulatory cells (Tregs) (42).

CTLA-4 is an ICP receptor primarily expressed on DCs and activated T cells including memory T cells and Tregs (8). It has two ligands, (B7-1) and CD86 (B7-2), mainly expressed on APCs. CD80 and CD86 also bind with a co-stimulation receptor, CD28, on T cells. Although CTLA-4 has been shown to have an intracellular working mechanism where binding with its ligands triggers downstream signaling (43, 44), the literature on CTLA-4 primarily focuses on its extracellular working mechanism. There therapeutic development around the CTLA-4 ICP also primarily leverages the extracellular mechanism. Extracellularly, CTLA-4 executes its immunosuppressive function by competing with CD28 for access to CD80 and CD86. One outcome of this competition is weakened proliferation and cytokine secretion by T cells (45). When CTLA-4 on Tregs binds with CD80 and CD86 on DCs, the DCs show reduced antigen-presenting capacity (46). CTLA-4 expressed on Tregs also augments the suppression effect of Tregs by extending the interaction time between Tregs and effector cells (47). Further, when mature DCs are treated with agonistic anti-CTLA-4 antibodies, the production of IL-8 and IL-12 by the DCs reduces by two thirds, and the antigen presentation ability of DCs decreases to approximately one fourth of the normal level. The treatment of the anti-CTLA-4 antibodies also weakened the capacity of the DCs to stimulate T cell proliferation by 50% (48, 49).

For the BTLA ICP, its receptor, BTLA, belongs to the CD28 family. BTLA is mainly expressed on activated T cells but also on resting B cells, mature DCs, and natural killer (NK) cells (9). The ligand of BTLA is the herpesvirus entry mediator (HVEM) that is expressed on resting T cells, macrophages, and immature DCs (17). In addition to BTLA, HVEM also binds with four other molecules: CD160 (expressed on effector T cells and NK cells), tumor necrosis factor superfamily member 14 (TNFSF14, also termed as LIGHT or CD258, expressed on activated T cells and immature DCs), lymphotoxin-α (expressed on T cells, B cells, and NK cells), and HSV-1 glycoprotein D (expressed on herpes simplex virus infected cells), independently. Indeed, HVEM is a “bidirectional switch” of immune activation (50–53). When HVEM binds with BTLA or CD160, immune inhibitory signals are transmitted (HVEM may also be referred to as a ligand of the CD160 ICP in this sense); when HVEM binds with TNFSF14, lymphotoxin-α, or HSV-1 glycoprotein D, immune stimulatory signals are transmitted. The engagement of HVEM by BTLA delivers a diminishing effect on immune stimulation. The engagement also dampens T cell proliferation and B cell activation, reduces the number of CD8+ DCs, and lessens the secretion of cytokines such as TNF-α, IFN-*γ*, IL-2, and IL-4 by these cells (52, 54–57).

Regarding the TIGIT ICP, the receptor, TIGIT, is expressed on Tregs, memory T cells, effector T cells, and NK cells after these cells are activated (10, 11). Up to now, TIGIT has been found to interact with three molecules: poliovirus receptor (PVR, also termed as CD155), PVRL2, and PVRL3. The immunological implication of the interactions between TIGIT and PVRL2 or PVRL3 remains elusive. The functional implication of the interaction between TIGIT and PVR is, however, much clearer and intriguing. PVR is found on DCs and B cells and has a high binding affinity to TIGIT. In addition to TIGIT, PVR also interacts with CD226 on naive T cells. When PVR binds with TIGIT on activated T cells, coinhibitory signals are transmitted (58, 59). The binding also dampens the proliferation and activation of T cells and promotes the generation of tolerogenic DCs (10). In contrast, when PVR binds with CD226 on naive T cells, costimulatory signals are delivered (58, 59). In this sense, PVR is a “bidirectional switch” like HVEM in the BTLA ICP. As for TIGIT on NK cells, its ligation with the PVR on B cells leads to a reduction of cytokine secretion by the NK cells. Also, the knockout of TIGIT from NK cells leads to a reduction in the IFN-*γ* secretion from the cells from 500 to 300 pg/ml in cell culture medium (60).

For the TIM-3 ICP, its receptor, TIM-3, is expressed on T cells, DCs, NK cells, and macrophages. There are at least four ligands identified for TIM-3: Galectin-9 (Gal-9), Phosphatidyl serine (61) high mobility group protein B1 (62) and Ceacam-1 (63). Among these ligands, Gal-9 is the best known and is ubiquitously expressed in the lymph nodes, spleen (18), and liver. Functional implications of the TIM-3 activation on Th1 cell, CD8 T cells, NK cells, macrophages, and DCs have been reported. The ligation of TIM-3 on Th1 cells by Gal-9 on hepatocytes decreases the secretion of cytokines such as IFN-*γ* and TNF-α by Th1 cells. The ligation also promotes apoptosis of Th1 cells (64), which is accompanied by an influx of calcium (65). The ligation of TIM-3 on CD8 T cells by Gal-9 leads to an inhibition of TCR signaling through the co-localization of TIM-3 and receptor phosphatases (66). For NK cells, it was proven that the blockade of TIM-3 on these cells boost the IFN-*γ* production by the cells (67). As for macrophages, the ligation of TIM-3 on these cells by Gal-9 on hepatocytes inhibits the production of IL-2 and IFN-*γ* by the macrophages (68). Last, the enhancement of the TIM-3 ICP on DCs by agonist antibody inhibits the activation and maturation of the DCs (12).

As for the LAG-3 ICP, its receptor, LAG-3, is expressed on activated T cells. It is also expressed on B cells, DCs, and NK cells. The ligand of LAG-3 is MHC class II on APCs. It was found that the ligation between LAG-3 and MHC II suppressed the proliferation of Th cells (12). In addition, Tregs are able to inhibit the activation and maturation of DCs through the LAG-3 ICP (69). And, the knockout of LAG-3 reduces the likelihood for CD4 T cells to differentiate into Tregs (70). As for NK cells, the LAG-3 knockout compromises natural killer activity in a mouse model (71). Lastly and interestingly, the PD-1 and LAG-3 ICPs show synergy with each other. Mice with the single knockout of PD-1 only show minimal autoimmune sequelae, such as a lupus-like condition. Mice with the single knockout of LAG-3 do not develop any autoimmune disorders within the first year after birth (72). In contrast, mice with the double knockouts of LAG-3 and PD-1 have lethal autoimmune disorders, including myocarditis and pancreatitis (73). In addition, two studies showed that cancer patients who were resistant to a single PD-1 ICP blockade therapy responded to the dual blockade therapy involving LAG-3 and PD-1 ICPs (74, 75).

CD200R1 is an ICP receptor that is expressed on resting macrophages, DCs, plasma cells, memory B cells, and T cells. T helper cells express a greater level of CD200R1 than cytotoxic T lymphocytes and naive T cells (19). The ligand of the ICP, CD200, is expressed on epithelial cells, endothelial cells, lymphoid cells, myeloid cells, neurons, and mesenchymal stem cells (19, 20). For macrophages and lymphocytes, the activation of the CD200 ICP boosts their production of TGF-β and IL-10, while decreasing their production of TNF-α, IL-1, IL-6, and INF-*γ* (20, 76–78). In addition, the activation of this ICP inhibits the activation of lymphocytes and macrophages, while promoting CD4 T cells to differentiate into Tregs (76–78).

VISTA is the newest ICP among the ones we discuss and was identified in 2011 (26). Its receptor, VISTA, is constitutively expressed on most immune cells except for B cells (13). VISTA is also known as a PD-1 homolog. The knockout of VISTA lowers the fraction of Tregs in the lung and spleen from 20% to approximately 10% (79). Since Tregs are critical to immune tolerance (80, 81), the reduction of Tregs implicates the importance of the VISTA ICP in maintaining immune tolerance. The VISTA ICP suppresses the proliferation and differentiation of T cells, as well as their production of cytokines such as IL-17 (82). The activation of the VISTA ICP results in the inhibition of the production of inflammatory cytokines and chemokines by DCs and macrophages, such as IP-10 and MCP-1 (83). To date, two ligands were reported for VISTA, V-set and immunoglobulin domain containing 3 (VSIG-3) and P-selectin glycoprotein ligand-1 (PSGL-1). VSIG-3 is expressed restrictively on neurons and glial cells in the brain and on Sertoli cells in the testis (21). Activation of the VISTA ICP by VSIG-3 retards the proliferation and cytokine secretion of T cells, such as the secretion of IFN-*γ*, IL-2, and IL-17 (84). PSGL-1 is expressed on many types of immune cells including T cells, B cells, DCs, and macrophages. It is also expressed on platelets and endothelial cells. The biological implications of the ligation PSGL-1 to VISTA remain elusive to date.

## ICP-Targeted Experimental Therapeutics for Autoimmune Diseases

**Figure 2:**
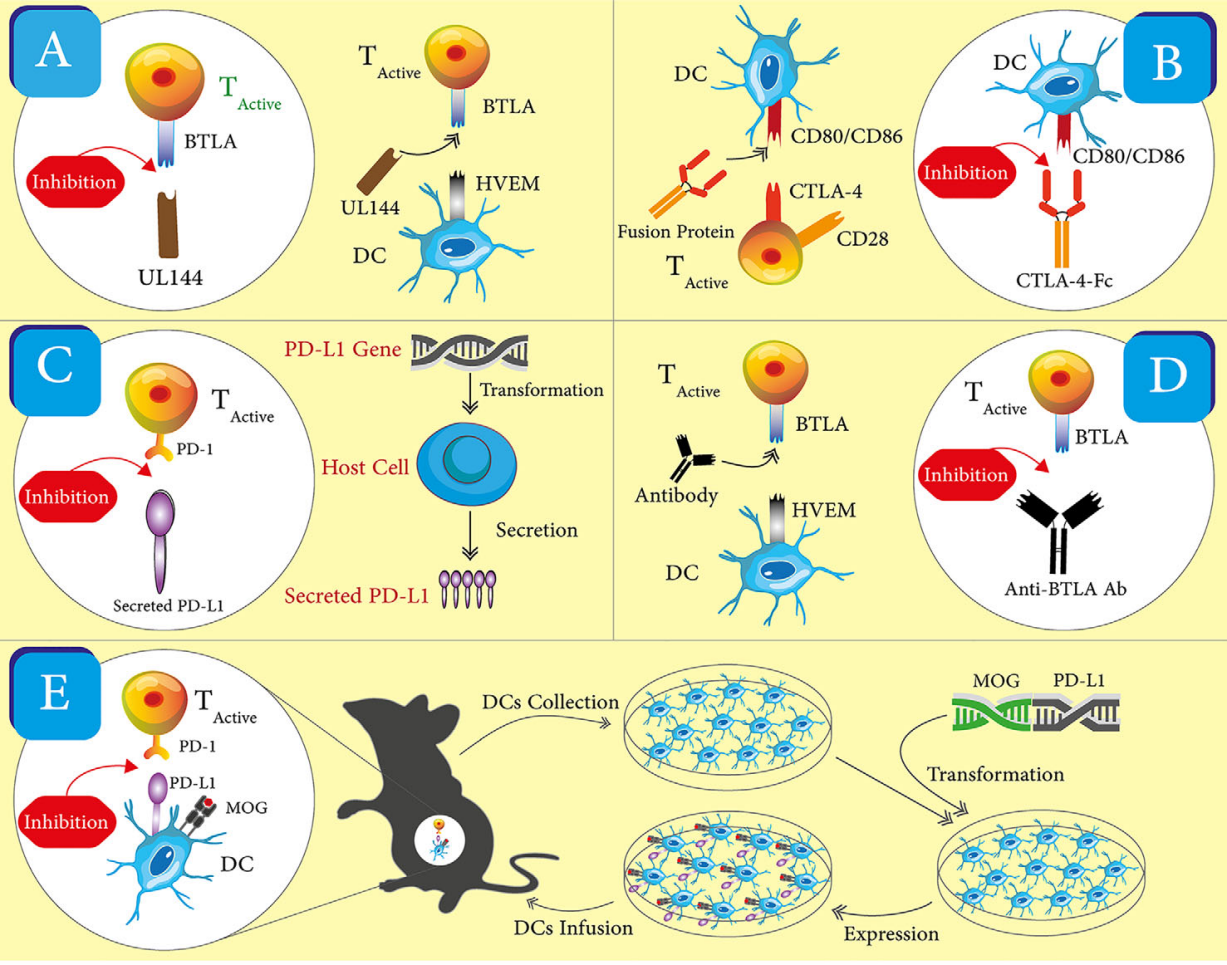
Schematics for the different forms of ICP-targeting therapeutics. One representative for each form of therapeutics was shown. **(A)** Viral proteins. UL144 is used to engage with BTLA and activate the corresponding ICP, which enhances immune inhibitory signals. **(B)** Soluble ligand and receptors. A fusion of CTLA-4 and Fc is used to engage with CD86/CD86 and activate the CTLA-4 ICP, which enhance immune inhibitory signals. **(C)** Nucleic acids. A coding gene of PD-L1 is used to increase the expression of PD-L1 in host cells. The increased expression strengthens the PD-1 ICP and immune inhibitory signals. **(D)** Antibodies. An anti-BTLA antibody is used as an agonist to enhance the BTLA ICP, which amplifies immune inhibitory signals. **(E)** Cells. DCs are collected and transfected with the coding genes of PD-L1 and MOG peptide. These engineered DCs have the enhanced expression of PD-L1 and MOG peptides. After these DCs are transferred back into mice, they promote immune inhibitory signals *in vivo* through the PD-1 ICP and the presentation of the MOG peptide to T cells.

The reported, ICP-targeted therapeutics may be divided into two classes based on their intended working mechanisms. The first class includes most of the reported therapeutics and shares a working mechanism—enhancing the ICP. This class takes the form of recombinant proteins, monoclonal antibodies, nucleic acids, and engineered cells (**Figure 2**). The second class embraces a more straightforward idea that ICP receptors or at least some ICP receptors on immune cells may be employed as biomarkers for pathogenic immune cells in autoimmune diseases. A corresponding working mechanism is the depletion of those ICP receptor positive cells. Currently, there is only one reported therapeutic in the second group. Below, we will recount these ICP-targeted therapeutics in the order of working mechanisms and forms. **Table 3** summarized these therapeutics and the disease models in which they are tested. We emphasize “experimental” in our section title because there is only one ICP-targeted drug, Abatacept, approved for use in clinics, which indicates both the challenges and opportunities in the development these therapeutics.

**Table 3:** Experimental therapeutics to enhance ICPs.

Therapeutic form	ICP	Disease models	Reference
Soluble ligand and receptor	PD-1	Lupus	(85)
		Experimental Autoimmune Glomerulonephritis	(86)
		Experimental Cerebral Malaria	(87)
		Psoriasis	(88)
	TIGIT	GVHD	(11)
	CD200		(89)
Viral protein	BTLA		(90)
Antibody	BTLA	AAV	(91)
	VISTA	GVHD	(92)
	VISTA	Experimental Asthma	(79)
Nucleic acid	BTLA	Herpetic Stromal Keratitis	(93)
	PD-1	Pancreatic Islet Transplantation	(14)
Cell	PD-1	EAE	(94)
	PD-1	Pancreatic Islet Transplantation	(95)
	CTLA-4	T1D	(96)
Combination therapy	CTLA-4 and BTLA	Pancreatic Islet Transplantation	(97)
Immunotoxin	PD-1	EAE & T1D	(36)

### Enhancement of ICPs

#### Soluble ICP Ligands and Receptors

Soluble ICP ligands are used to enhance the activity of an ICP. For example, soluble PD-L1 was used to engage with PD-1 and flick on the PD-1 ICP. On the other hand, soluble ICP receptors were used in a more fascinating manner. Taking the TIGIT ICP as an example, TIGIT, as a receptor, engages with its cognate receptor, CD155, on DCs (10). The engagement dampens the activation signaling mediated by CD226 because the activation signaling depends on the interaction between CD155 and CD266 while TIGIT’s “occupation” of CD155 prevents the interaction (58, 59). Soluble TIGIT also has the ability to prevent the interaction between CD155 and CD266.

The developers of these soluble ICP ligands and receptors have put them on a proven protein “Noah’s Ark”, the fragment crystallizable region of an antibody (Fc). When fused with Fc, proteins usually assume longer *in vivo* half-lives. Fc also helps to immobilize Fc fusion proteins on the cell surface of APCs through binding with Fc receptors on these cells (98). These fusion proteins are often produced as secreted proteins using a mammalian cell expression system. One example of such production procedure is that Wang and coworkers generated an expression plasmid carrying the coding genes for the mouse PD-L1 extracellular domain and the mouse lgG1 Fc (87). Then, they transfected FreeStyle 293 cells with the plasmid. Lastly, they purified fusion proteins from the supernatant of the cell culture.

The most successful soluble ICP receptor-based therapeutic is Abatacept. Abatacept is a fusion protein that consists of the extracellular domain of human CTLA-4 and the Fc of human IgG1 (99). Compared to CD28, Abatacept has a greater affinity to CD80 and CD86. Abatacept is able to inhibit the CD28-mediated stimulation signaling through competition for CD80 and CD86. The therapeutic has a plasma half-life of 14.7 days in patients in the dosing range of 0.5 to 50 mg/kg (99). Abatacept was approved to treat RA. Arthritis patients were evaluated based on a 50% improvement in the number of tender and swollen joints (American College of Rheumatology, ACR50) after treatments. Among Abatacept-treated patients, 21.8% of reached ACR50 after 6 months; in contrast, only 3.8% of placebo-treated patients reached ACR50 (100). The same study also proved that this medicine is safe: adverse events happened to 79.5% of the Abatacept-treated patients and 71.4% of the placebo-treated patients; but there is no increase in serious infections among Abatacept-treated patients when the infections of Abatacept-treated and placebo-treated patients were compared (100). In 2011, a derivative of Abatacept, Belatacept, was also approved to be used as an immunosuppressive agent for kidney transplantation.

Besides Abatacept, other PD-L1-Fc fusions have also been reported (86–88, 101). The efficacy of these fusions was demonstrated in various autoimmune disease models, including experimental autoimmune glomerulonephritis and lupus. Zhou et al. found that PD-L1-Fc significantly reduced the auto-antibody production and tubular proteinosis in murine lupus models according to histological assessments. The treatment of PD-L1-Fc extended the survival of treated mice from an average of 45 to 60 weeks. When PD-L1-Fc was used in a prophylactical manner, it completely prevented proteinuria (101). Reynolds and coworkers showed the treatment of PD-L1-Fc reduced the glomerular infiltration of T cells by half in a mouse experimental autoimmune glomerulonephritis model (86). It is noteworthy that PD-L1-Fc fusions have also demonstrated their ability to weaken immune responses in inflammation and infection models. A study reported by Wang et al. showed that PD-L1-Fc reduced the number of CTLs by 75% and alleviated the disruption of the blood–brain barrier that was caused by over-reactive CD8 T cells in the mouse experimental cerebral malaria model (87). The treatment also increased the survival rate of the mice from 10 to 60% by 10 days after infection. Kim and coworkers demonstrated that the treatment of PD-L1-Fc reduced the production of IL-17A by T cells by 75% and alleviated psoriasis inflammation in imiquimod-treated mice (88). Together, these data suggest that PD-L1-Fc is able to suppress immune responses in both autoimmune disease and inflammation models.

The TIGIT-Fc fusion is an example of soluble receptors that are used to compete for its cognate receptors. The fusion consists of the extracellular domain of mouse TIGIT and human lgG3 Fc domain. The TIGIT-Fc was found to inhibit the activation of macrophages and increase their secretion of IL-10, an anti-inflammatory cytokine, by three times (102). Further, in the mouse acute GVHD model, the treatment of TIGIT-Fc increases the survival rate from ~5 to ~30%. Among the mice that have already shown the GVHD symptoms, TIGIT-Fc prolongs their survival time from shorter than 30 days to longer than 40 days (11). Together, these data validate the idea that TIGIT-Fc can be used to suppress immune responses.

There was also an exploration of adding a cytokine into the fusion proteins that consist of ICP ligands and the Fc (89). Gorczynski and coworkers generated an immunosuppressive protein that includes CD200, Fc, and TGF-β. There is a Glycine (n = 6) linker between the Fc and TGF-β (93). The fusion protein, CD200FcGly6TGF-β; was able to bind with TGF-β receptors on T cells and CD200R1 on APCs. The immunosuppression effect of the protein was observed both *in vitro* and *in vivo*. Specifically, the treatment of CD200FcGly6TGF-β boosted the percentage of Tregs in the spleen from ~3% to more than 10%.

#### Viral Proteins

Some viral proteins mimic the biological functions of ICP receptors or ligands and are hence explored as an “Off” switch for undesirable immune responses (90, 103). UL144, a cytomegalovirus protein, is an ortholog of HVEM, a ligand of the BTLA ICP (104). However, in contrast to HVEM that interacts with not only BTLA but also CD160, TNFSF14, lymphotoxin-α, and HSV-1 glycoprotein D (50), UL144 only binds with BTLA. Thus, while HVEM is a bidirectional switch depending on its binding partners, UL144 is dedicated to activate the BTLA ICP (105). In addition, UL144 is approximately three times more potent than HVEM in suppressing the proliferation and activation of T cells, despite the fact that UL144 binds five times weaker to BTLA than HVEM (90). Further, the binding selectivity of UL144 was attributed to its N-terminal cysteine-rich domain 1 (CRD1) and the CRD2 loop (106). Sedy and coworkers leveraged the binding selectivity insight of UL144 and engineered a tetra-mutant of HVEM that carried four site mutations, S58R, G68T, L70W, and L90A. The tetra-mutant of HVEM not only assumed the binding selectivity just like UL144 but also acquired a 10-fold stronger affinity to BTLA compared with the wild type (90). The tetra-mutant exhibited a significant inhibitory effect to the SHP-1-sensitive, type I interferon signaling pathway of B cells and NK cells. It also effectively inhibited TCR-mediated antigen signaling.

#### Monoclonal Antibodies

Monoclonal antibodies are one of the most common forms of therapeutics in general. They have specific binding target and activity, and possess desired safety and pharmacokinetics properties. Their popularity is also backed by robust antibody engineering and production technology, which ensure reproducible and economically sensible products. Thanks to these advantages, monoclonal antibodies are also a popular form of therapeutics that enhance ICPs even though they were not examined exclusively in autoimmune disease models. Monoclonal antibodies are developed as agonists of ICP receptors to enhance immune regulation. It is noteworthy that along with the development of ICP-enhancing antibodies, there are successful developments of monoclonal antibodies as ICP blockers. These blockers are developed to facilitate immune stimulation and are now exclusively used in boosting anti-cancer immune responses. Seven of such ICP blockers have been approved by the FDA for cancer therapy. This development outcome, on the one hand, highlights the feasibility to develop antibody-based therapeutics to target ICPs, but on the other hand, alerts us to distinguish ICP-blockade and -enhancement antibodies.

An anti-BTLA antibody was found to have the ICP agonistic effect in the antineutrophil cytoplasmic antibody-associated vasculitis (AAV) model (91). This antibody suppressed T-cell proliferation by 32% and IL-17A secretion by 30% in the AAV model. It is worth mentioning that IL-17A is a key driver in pathogenesis of AAV and that the knockout of IL-17A protects mice from developing AAV (107, 108). IMP761, a humanized anti-LAG-3 antibody, showed immunosuppressive effect in a delayed-type hypersensitivity model of cynomolgus macaque that was induced by tuberculin. IMP761 decreased the infiltration of inflammatory T cells into the DTH site to one third of the control level. *In vitro*, IMP761 reduced the proliferation and activation of self-antigen-induced T cells with a mean inhibition rate of 50% (109).

The immunosuppressive effect of ICP agonist antibody was also demonstrated in the graft-versus-host disease (GVHD) and asthma models. Dallas and coworkers showed the treatment of an anti-VISTA antibody extended the survival of mice of the GVHD model. Treated mice were able to live up to 18 months with no sign of GVHD, infection, or cancer (92). Meanwhile, the treatment reduced the accumulation of T cells in the spleen and liver by eight and five times, respectively. When an anti-VISTA antibody was used to treat OVA-induced asthma in mice, the accumulation of eosinophils in bronchoalveolar lavage fluid of these mice was reduced to one third of the untreated, control level (79). According to the histological analysis, the mucus production also decreased significantly in the antibody-treated mice. Data from these aforementioned animal models reinforces the point that agonistic antibodies of ICPs could be a powerful tool to enhance ICPs and suppress autoimmunity and other undesired immune responses.

It is noteworthy that the sub-classification of agonist antibodies influences their efficacy (110). lgG is the most popular class of therapeutic antibodies due to its stability and long half-life. Among the subclasses of IgG, IgG2 appears to be the most effective subclass of agonists (95, 111–113). When three subclasses anti-CD200R antibodies, lgG1, lgG2, and lgG4, were compared, lgG2 showed 2-fold greater efficacy than both lgG1 and lgG4 (110). This result suggests the importance of considering the subclasses and the Fc structure of antibodies when designing antibody therapeutics targeting ICPs.

#### Nucleic Acids

Genes that encode ICP receptors and ligands are used as therapeutics to suppress immunity. These genes are able to increase the production of receptors and ligands inside the body. Plasmids and virus are used as vectors to deliver the genes and transfect cells in murine models. To date, this gene therapy approach has achieved an increased expression of receptors and ligands as well as immunosuppression in the animal models of inflammation and organ transplantation. It could be effective in autoimmune disease models.

A plasmid that carries a coding gene of BTLA was used to treat herpetic stromal keratitis (93). According to immunohistological analysis, this treatment decreased the infiltration of CD4 T cells into infected corneas by half. The treatment also reduced IFN-*γ* positive cells in murine corneas and draining lymph nodes by seven and two times, respectively. Further, splenocytes collected from the treated mice produced less IFN-*γ* (0.4 ng/ml versus 1.4 ng/ml of the control group). The result of IFN-*γ* is a sign of weakened Th1 responses. Ultimately, the treatment with the BTLA plasmid lowered the incidence rate and the severity of corneal lesions (the clinical score decreased from ~4 to less than 1, even 0).

An adenovirus was constructed to deliver the coding gene of PD-L1 by Li and coworkers (14). The adenovirus was preferred for gene delivery due to their high transfection efficiency and low toxicity. This PD-L1 adenovirus was tested in a pancreatic islet transplantation model using mice with diabetes. The treatment with the virus resulted in a high-level presence of soluble PD-L1 in mice for at least 28 days (21 mg/ml in caudal vein blood). The treatment also prolonged the survival time of islet grafts from an average of 8 to 28 days. In addition, the treatment lowered blood glucose levels (from >20.0 mmol/L to <11.1 mmol/L), another indicator that the treatment helped to protect islet grafts.

#### Cells

Since ligands of ICPs are intrinsically expressed in hematopoietic and non-hematopoietic cells to ignite ICPs, it is a reasonable strategy to supplement cells that express the ligands to the body in order to strengthen ICPs. DCs express multiple types of ICP ligands (12, 14, 16, 46, 84). and hence were exploited to carry out the task. Hirata and coworkers used this approach to prevent EAE (94). They engineered DCs that co-expressed both PD-L1 and a myelin oligodendrocyte glycoprotein-derived antigenic peptide (MOG, p35-55). The mice that received a transfer of these dual expression DCs experienced the significantly reduced severity of EAE as compared to untreated mice or mice that were treated with DCs expressing the MOG peptide only. The mean clinical score reduced from 3.0 to less than 1.0. Treatment with the PD-L1/MOG peptide dual expression DCs also abolished T cell infiltration into spinal cords. As a prophylactic measure, the dual expression DCs prevented the development of EAE and maintained a mean clinical score under 1.0. It is worth mentioning that PD-L1 and the MOG peptide are preferred to be expressed by the same DCs according to data. When PD-L1 and the MOG peptide are expressed in two separate DC populations, and when the combination of the two populations was used to treat mice with EAE, the combination therapy did not deliver the same level of efficacy as the dual expression DCs. The result underscores the necessity of co-expression of PD-L1 and the MOG peptide in the same DCs for this therapeutic strategy.

In addition to DCs, other types of cells were also engineered and utilized to ameliorate autoimmune diseases. Pancreatic β cells were engineered to express the scFv of anti-CTLA-4 antibody (96). In a coculture experiment containing T cells and the engineered β-cells, the β-cells inhibited the level of T cell proliferation by half. The same research group also generated transgenic NOD mice that specifically express the scFv of anti-CTLA-4 antibody in β cells. The expression was induced by insulin. It was found that, at 21-weeks old, the diabetes incidence rate for the wile type NOD mice was 59%, while the rate for the CTLA-4 expression NOD mice was only 7.4%.

The cell engineering strategy was also employed to protect transplanted organ allografts. Wen et al. generated pancreatic β-cells that overexpress PD-L1 (95). They treated streptozotocin-induced diabetic mice by intraperitoneal injection of allogenic PD-L1-overexpressing β-cells. The engineered β-cell allograft had much longer survival than the wildtype β-cell allograft, more than 60 days versus fewer than 20 days. Their analysis further showed that the engineered β-cells lowered lymphocyte proliferation by half, increased the apoptosis rate of lymphocytes from ~40 to ~60%, and reduced the secretion of IFN-*γ* by lymphocytes from ~25 to ~10 pg/ml.

#### Combination of Therapeutics

Since some of the aforementioned therapeutic strategies have complementary working mechanisms, it is reasonable to explore combination therapies that consist of multiple of strategies. One reported effort is the combination of soluble ICP molecules and antibodies (97). Truong et al. designed a combination that included an anti-BTLA antibody and a CTLA-4-Fc fusion and used the combination to foster immune tolerance toward islet allografts in mice (97). This combinational treatment led to indefinite allograft survival (>100 days) through attenuating CD4 and CD8 T-cell mediated immune rejections. In contrast, the monotherapy of the anti-BTLA antibody or the CTLA-4-Fc fusion only delivered moderate survival benefits; the islet allografts survived for 20–30 days.

### Depletion of ICP Receptor Positive Immune Cells

Existing data on the PD-1 ICP point to the importance of PD-1 positive cells in the pathogenesis of multiple autoimmune diseases including T1D, MS, and SLE (29, 114–122). Further, a correlation was reported between the pathogenic potential and the PD-1 expression of autoreactive lymphocytes (123). Thus, it is plausible to use PD-1 as a biomarker of autoreactive immune cells in autoimmune diseases. We recently created an immunotoxin that is able to specifically ablate PD-1 positive cells (36). The depletion of PD-1 positive cells effectively ameliorated autoimmune attacks. Specifically, the depletion treatment delayed the onset age of the spontaneous T1D in NOD mice from 19 to 29 weeks. The depletion also enabled mice to recover from the advanced stage of EAE. What makes this depletion treatment even more desirable is that the treated mice maintained healthy adaptive immunity, evidenced by their full-strength humoral and cellular immune responses to vaccinations. The healthy adaptive immunity was protected after the depletion of PD-1 positive cell because naive lymphocytes, which are PD-1-negative (PD-1^−^), are spared by the depletion (15, 124–126). These naive lymphocytes can be activated, mount normal immune responses, and maintain healthy adaptive immunity after the depletion.

## Safety of ICP Related Autoimmune Therapies

Therapeutics for autoimmune diseases, in principle, should exert an immunosuppressive effect. Often, such suppression happens to not only autoimmunity but also healthy immunity, leading to immune deficiency and undesirable side effects. Patients who take these therapeutics long term are prone to opportunistic infections and endure a greater risk of malignancy. Thus, it is critical to evaluate whether autoimmune disease therapeutics cause unacceptable immune deficiency during the therapeutic development. Such a requirement should apply to ICP-based therapeutics as well.

According to reported data of ICP-based therapeutics, these therapeutics appear devoid of serious side effects. None of the cited studies in this review revealed serious side effects. Given that the efficacy evaluation of these therapeutics normally requires long-term studies, it is reasonable to assume these therapeutics do not cause severe side effects within a reasonably long period of time. In our recent study, we continuously applied the treatment of PD-1 positive cell depletion to NOD mice and observed these mice for 17 weeks. We did not observe any serious side effects on the mice during the entire study except for hyperglycemia at the end phase of the study (36). In Zhou’s study of PD-L1 fusion proteins, treated mice were followed for more than 60 weeks after treatment. No severe side effect was detected (101). In addition, Abatacept was proven safe for clinical use through clinical trials (127). These results may serve as an indication that these ICP therapeutics are devoid of severe side effects when treating autoimmune diseases.

In addition to the aforementioned passive observations of side effects, some studies of ICP-based therapeutics included an active assessment of protective immunity after treatments. In the above-mentioned PD-1 positive cell depletion study, we evaluated blood cell counts and humoral and cellular immunity two days after the depletion. Our data showed that treated mice have a normal level of B cell, CD4 T cell, and CD8 T cell counts in blood and spleens (36). These treated mice developed normal antibody and cytotoxic T lymphocyte (CTL) responses toward vaccinations. In a study of PD-L1-Fc proteins, the administration of PD-L1-Fc did not alter the percentages of peripheral CD4 and CD8 T cells, nor the population of FoxP3 positive cells (101). However, the treatment of PD-L1-Fc reduced the fractions of antibody-secreting cells among peripheral mononuclear cells, splenic cells, and bone marrow cells to one third of the normal levels. Together, these results of active assessments suggest that the impact of ICP-based therapeutics on protective immunity may be dependent on the mode of action of these therapeutics. Meanwhile, while the side effects of ICP therapeutics are generally mild, it is difficult to draw general conclusions about their safety because of their distinct working mechanisms. Meanwhile, these results are promising because they suggest the possibility to mitigate side effects of ICP therapeutics by employing therapeutics with different mode of actions.

## The Prospect of ICPs as a Class of Therapeutic Target

ICPs have promising prospects as targets for autoimmune disease therapeutics. Such a point of view is backed by various facts and reasonings that we will state below. These facts and reasonings may be categorized into four aspects, clinical successes, mechanistical support, preclinical successes, and safety assurance of ICP-based therapeutics.

First and foremost, there is already an FDA-approved drug targeting an ICP. Abatacept targets the CTLA-4 ICP and was approved as a treatment of a type of RA that does not respond to disease-modifying anti-rheumatic drugs and anti-TNF-α. Besides Abatacept, an anti-PD-1 agonist antibody (Celgene: CC-90006) has been tested clinically in psoriasis patients since 2016 (NCT03337022). In all, although there is only one FDA-approved ICP drug thus far, the clinical progress still underscores the feasibility of developing autoimmune disease therapeutics targeting ICPs.

Second, there exists a wide range of data supporting ICPs as the targets for autoimmune disease therapeutics from a mechanistic aspect. Data from the murine models demonstrated the critical relationship between the dysfunction of ICPs and the pathogenesis of autoimmune diseases (23, 29–36, 128). Further, clinical data revealed the correlation between autoimmune diseases and the deficiency of ICP signaling in humans (129–133). These data, together, show the important role of ICPs in the maintenance of immune tolerance and support the notion that molecules that restore or enhance ICP signaling could be effective therapeutics to prevent, alleviate, or cure autoimmune diseases.

Third, the experimental therapeutics we recounted in Section 3 showed solid efficacy in various animal models of autoimmune disease and inflammation (**Table 3**). These therapeutics are able to suppress autoimmunity and inflammation. These preclinical results validate ICPs as an appealing therapeutic target for the treatment of autoimmune diseases. In addition, these results confirmed that fairly diversified methods and agents can be used to leverage on ICPs to dampen autoimmunity.

Last, experimental therapeutics that target ICPs showed relatively mild and potentially acceptable side effects. As summarized in Section 4, no severe side effect was reported for the wide range of therapeutics we reviewed. Although one therapeutic of one ICP was shown to impair humoral responses, another therapeutic targeting the same ICP but having a different mode of action did not compromise humoral responses. While more systemic studies are required to draw a general conclusion on the safety of the therapeutics that target ICPs, the safety data to date point to a positive prospective for these therapeutics.

Overall, the development of ICP-targeted, autoimmune disease therapeutics is still in its infancy and trails behind the development of ICP-targeted cancer therapeutics: fewer drugs were approved for clinical use; fewer publications are present. The underdevelopment of autoimmune disease therapeutics could be attributed to multifaceted factors: greater research efforts and investment in cancer therapy development, the urgency to develop cancer therapeutics, the higher safety requirements for autoimmune disease therapeutics, and so on. The underdevelopment reality, however, also provides a broader horizon to explore ICPs as therapeutic targets and utilize the targets. There is a great development space to explore. Given the aforementioned prospects of this class of therapeutics, it is reasonable to believe that ICPs as therapeutic targets will yield more successes in the future.

## Conclusion

ICPs are an important mechanism to maintain immunotolerance and suppress autoimmunity. Evidences from preclinical studies and clinical trials have shown that an abolishment or a blockade of ICPs leads to the loss of immune tolerance and autoimmune disorders. To date, there are eight ICPs that have been mostly studied and reported. Distinct to other reviews of ICPs that focus on their utilizations in cancer immunotherapy (85, 134, 135), this review focuses on autoimmune disease therapeutics targeting ICPs. These therapeutics may be categorized into two groups according to their working mechanisms: the first group shared a working mechanism of enhancing ICPs; the second group utilized ICP receptors as biomarkers for pathogenic immune cells of autoimmune diseases and treated the diseases by depleting ICP-receptor positive immune cells. The ICP-based autoimmune disease therapeutics, in general, have acceptable safety profiles although more clinical and long-term evaluations are warranted. Given the impact of the ICPs on immune tolerance and the current development status of ICP-based therapeutics, it is deemed that there is a great space to develop this line of therapeutics, and this type of therapeutics holds promise to transform autoimmune disease therapy.

## Author Contributions

YZ and MC contributed the idea of this manuscript. All authors contributed to the article and approved the submitted version.

## Funding

MC is supported by the funding from the NIAID and NMSS. This work was supported by the NIH, 1R01AI139535 and National Multiple Sclerosis Society, GR-1807-31630.

## Conflict of Interest

The authors declare that the research was conducted in the absence of any commercial or financial relationships that could be construed as a potential conflict of interest.
